# Scope and financial impact of unpublished data and unused samples among U.S. academic and government researchers

**DOI:** 10.1016/j.isci.2023.107166

**Published:** 2023-06-19

**Authors:** Emma C. Bowers, Jimena Stephenson, Melissa Furlong, Kenneth S. Ramos

**Affiliations:** 1 LabPair, Inc., Tucson, AZ, USA; 2 University of Arizona Mel and Enid Zuckerman College of Public Health, Department of Community, Environment, and Policy Tucson, Tucson, AZ 85724, USA; 3 Texas A&M Institute of Biosciences and Technology, Center for Genomic and Precision Medicine, Houston, TX 77030, USA

**Keywords:** Biological sciences research methodologies, Surveying, Research methodology social sciences

## Abstract

Unpublished data and unused samples are common byproducts of research activity, but little is known about the scope and economic impact of their disuse. To fill this knowledge gap, we collected self-reported anonymous survey responses from 301 academic and government scientists from randomly selected institutions. Respondents estimated that they published ∼60% of their data and 95% had unpublished data. Of those collecting specimens, 60% stored unused samples. Systemic and logistical issues were identified as major contributory factors. The median cumulative self-reported estimated value of unused resources per researcher was $28,857, with life science ($36k) and government ($109k) researchers reporting the costliest assets. Using NSF headcounts, we estimated that the current cumulative value of unused resources at universities is approximately $6.2 billion, about 7% of the current annual R&D budget. These findings provide actionable information that can be used by decision makers to reduce obstacles that undermine scientific progress and productivity.

## Highlights

• Unpublished data and unused samples represent billions of dollars in stranded assets
• Unfinished projects and orphan data were the primary sources of unpublished data
• Unused samples are not shared because others don’t know they exist
• Addressing logistical problems can increase the efficiency of research

## Graphical abstract

**Figure:**
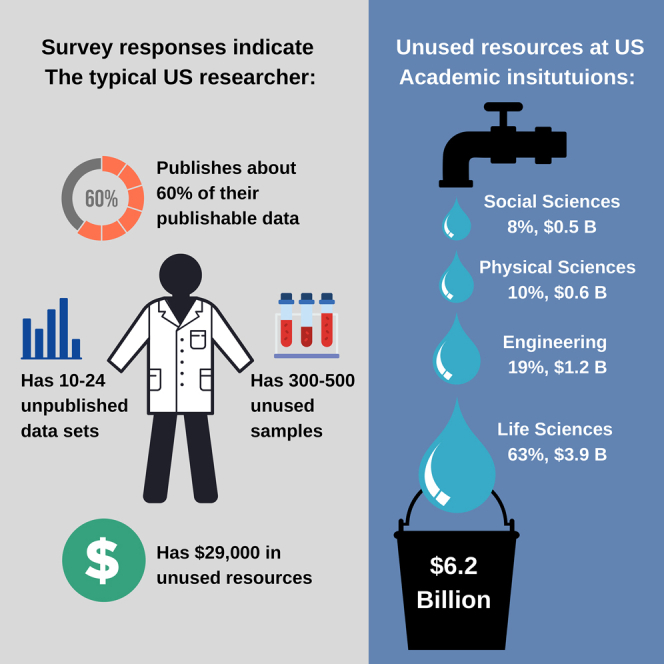

## Introduction

In the United States over $600 billion is funneled into R&D each year to advance scientific knowledge and to address pressing societal needs.^1^ Despite efforts to guarantee a return on investment through mandated reporting and specified deliverables, the problem of unpublished data and unused specimens remains. Ironically, while unpublished data are almost universally acknowledged by scientists, relatively few studies have examined their economic impact or the reasons for their disuse.

Some scientists have been quite vocal about the inefficiency in research.^2^^,^^3^^,^^4^^,^^5^^,^^6^^,^^7^^,^^8^^,^^9^ These concerns reached a pinnacle in 2009 when Iain Chalmers and Paul Glasziou estimated that over 85% of biomedical research is avoidably wasted.^10^ Marija Purgar et al.^11^ arrived at a similar estimate for ecology. Although for both analyses this waste was attributed to a variety of factors, including flaws in relevancy, design, methodology, bias, etc.; lack of publishing and reporting of data were one of the main causes.^6^^,^^9^

To date, most of the public discourse on unpublished data have been field-specific^7^^,^^11^^,^^12^ with a central focus on highly visible biomedical studies such as clinical trials. However, it has been deduced that most “dark data” are actually held by a greater number of small labs receiving less sizable grants.^8^ In addition, unused samples have yet to be considered in this problem. To begin to address this knowledge gap, we collected anonymous information on unpublished data and unused samples from 301 US scientists representing a breadth of fields, institutional types and sizes, and research roles. Our goal was to quantify the amount of unpublished data and unused samples researchers possessed and understand the reasons for their disuse.

Recent changes in publication practices have expanded what it means to “publish” data, thus, we established clear definitions for what constituted “unpublished data” (Table 1). Publishing data ahead of peer-review (pre-prints) and uploading stand-alone data in repositories is a valuable way to promote data sharing among scientists and overcome many limitations of the peer-review publication system.^13^^,^^14^^,^^15^^,^^16^ Despite promising gains in popularity, widespread adoption has yet to be achieved and varies considerably by field^17^^,^^18^^,^^19^ with researchers and publishers debating how this information should be used and presented.^20^^,^^21^^,^^22^^,^^23^ In the mean-time peer-reviewed manuscripts are still viewed by many as the “gold standard” for justifying experimental rationale and demonstrating productivity for grants and career advancement. Thus, we retained the traditional view of “publishing” as being in peer-reviewed scientific journals. However, many pre-prints published ahead of journal submission would be included in our definition of “publishing” and using these new mechanisms does not prevent data from being published in peer-reviewed format. Study definitions are presented in Table 1.

**Table 1:** Core definitions as provided in survey

Terms	Definition as provided to respondents
Unpublished Data	“Publishable” data that have not been published in a peer-reviewed scientific journal, are not in articles currently under review, and are not a part of a publication to be submitted in the coming year.
Publishable Data	Data that are suitable for publication in a peer-reviewed scientific journal because they meet rigor and reproducibility standards in your field. Do not exclude data from your estimates based on their projected value or impact (i.e. negative or inconclusive results)
Negative Results	Publishable data in which there is a failure to reject the null hypothesis (i.e., an effect doesn’t happen). For negative results to be publishable, the proper positive and negative controls must be included and there should be no technical or experimental issues.
Orphan Data	Data that doesn’t “fit” well into the lab’s other papers but does not constitute an entire publishable unit
Ancillary Findings	Findings unrelated to lab’s mission
Publication Efficiency	100% Efficiency = Publishing 100% of all publishable data in peer-reviewed journals
1 Unit of Data	Sufficient data to create a single graph/table.
Unused Samples	Samples that are produced in excess, left-over from experiments or collections, or so easily generated that the laboratory would be willing to share them with other respected collaborators. DO NOT include Precious samples that the laboratory would be unwilling to share; Samples/specimens that are not suitable for use in publication due to insufficient rigor or reproducibility standards or technical/experimental issues.

## Results

### Respondent demographics

To assemble contact lists for academic institutions, a random number generator was used to select institutions that were stratified by Carnegie Classification^24^ and sector. Carnegie Classification ranks accredited degree-granting institutions by factors such as the size of the student body, degrees conferred, and/or research dollars. We selected 22 R1 (doctoral universities—very high research activity), 15 R2 (doctoral universities—high research activity), 15 D/PU (doctoral/professional universities, moderate research activity), and 15 M1-3 academic institutions (Master’s colleges and universities; 1, larger; 2, medium; and 3, smaller programs) and 28 government research agencies that represented 39 states across the US. Contact lists were compiled from departmental websites or institutional directories and primarily included tenured and tenure-track faculty. Research staff, post-docs, and graduate students were also included if their contact information was available.

Survey invitations were sent to 10,206 contacts via email and an additional 176 contacts made via LinkedIn messages Invitation emails from 568 accounts bounced due to defunct email addresses or recipients opting out of Survey Monkey emails. Of the 5,713 individuals who opened their invitation emails, 317 responses were received for a 5.5% response rate and a 74% completion rate. The survey questions can be found in Data S1.

Sixteen responses were excluded from further evaluation due to incomplete information. The remaining 301 respondents were binned by research field into life sciences (54%), physical sciences (24%), social sciences (15%), or engineering (7%). Position and sector data sums exceeded 100%, as some respondents held cross-institutional appointments. The majority of respondents were academic researchers affiliated with R1 institutions (58%), followed by R2 (21%), and government researchers (14%). Researchers at D/PU and M1-3 institutions were collapsed into a single category (11%) due to low numbers. Most respondents were tenured faculty (44%), tenure-track faculty (14%), or non-tenure track faculty/postdocs (23%). Other position and demographic information are indicated in Table 2.

**Table 2:** Respondent demographics

Respondent characteristics	Count	Percent
**Gender**		

Male	179	59%
Female	109	36%
Non-Binary	1	0%
Prefer not to answer	12	4%
Total	301	100%

**Field**		

Life Sciences	164	54%
Physical Sciences	71	24%
Engineering & Technology	20	7%
Social Sciences (Psych, Sociology, Anthrop)	46	15%
Total	301	100%

**Sector/Tier**^a^		

Government	43	14%
Academia - R1	174	58%
Academia - R2	63	21%
Academia - D/PU, M1-M3	33	11%
Industry	2	1%
Non-Profit	7	2%

**Position**^a^		

Tenured Faculty	133	44%
Tenure-Track Faculty	41	14%
Government Scientist	43	14%
Non-Tenure Track Faculty and Postdocs	68	23%
Graduate Students	22	7%
Lab Managers	4	1%
Retired	5	2%
Physicians	17	6%
Veterinarians	5	2%
Industry Scientist	2	1%

a Some individuals held dual affiliations and/or job titles.

### Unpublished data

Respondents were asked detailed questions regarding their unpublished data, as defined in Table 1. The publication process is a lengthy continuum that can dead-end in the manuscript preparation or revision stage.^7^^,^^12^ To institute a reasonable time cutoff, we asked researchers to exclude from their responses data that were “part of articles currently under review and/or not part of a publication to be submitted in the coming year” (which would include many pre-prints). Finally, we asked respondents to remain unbiased as to the data’s projected value or impact (i.e., not to exclude data in which the results were negative, null, or inconclusive). Additional definitions used in the study are presented in Table 1.

**Figure 1:**
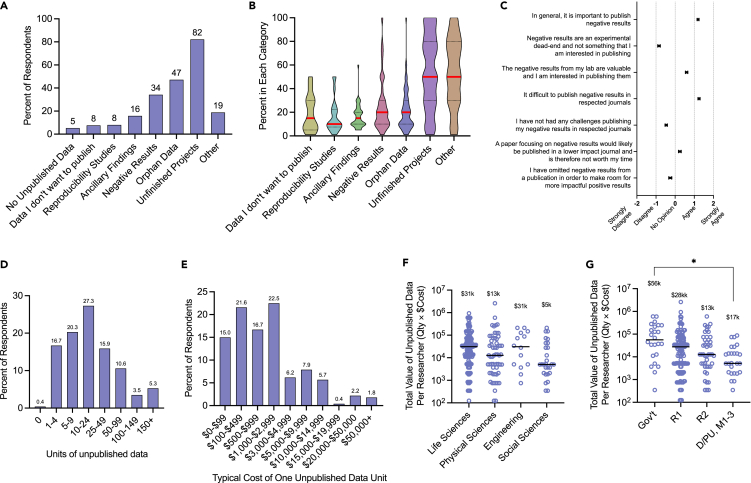
Self-reported estimates of the quantity, type, and cost of unpublished data among researchers Unpublished data were defined as being: publishable (meets respondents’ field’s rigor and reproducibility standards; no technical issues); not currently published in a peer-reviewed scientific journal; not in articles currently under review; not part of a publication to be submitted in the coming year. Respondents were asked to remain agnostic of projected value or impact. (A) Percent of researchers possessing each type of unpublished data. Types of data meriting additional definitions: Ancillary findings, data unrelated to the lab’s mission; Orphan data, data that does not “fit” well into the lab’s other papers but does not constitute an entire publishable unit; and negative results, data in which there was a failure to reject the null hypothesis, includes positive and negative controls, and was collected without technical issues. (B) Violin plot depicting the percent of researchers’ unpublished data falling into each category. Median indicated by red line. (C) Inventory of attitudes and experiences regarding the publishing of negative data. Mean and s.e.m. shown. (D) Percent of respondents possessing indicated quantities of unpublished data and (E) Percent of respondents indicating average cost of producing one unit of data (excluding employee time cost). (F and G) Calculated estimate of unpublished data per researcher (avg quantity × avg cost) stratified by field (F) and sector/tier (G). Median indicated by solid lines and annotations. ∗p < 0.05, One-way ANOVA of log transformed values with Tukey’s multiple comparisons. Mean and s.e.m. shown. *n* = 240–301.

Based on the criteria given, 95% of respondents indicated that they possessed unpublished data. Examples of responses provided included: “gene overexpression in cell lines,” “plant physiology,” “training evaluation data,” “large longitudinal dataset of pregnancy,” “imaging studies on human brain tissue,” “attitudes and perceptions on local environmental issues,” “diversity and prevalence of pathogens,” “sediment transport to marshes,” etc. (Data S2). Respondents were then asked if they possessed unpublished data falling into certain categories (Figure 1A) and to estimate how much of their unpublished data fell into each of those categories (Figure 1B). Types of data meriting additional definitions included: ancillary findings, data unrelated to the lab’s mission; orphan data, data that did not “fit" well into the lab’s other papers but would not constitute a publishable unit; and data producing negative results (also termed “null data”), data for which there was a failure to reject the null hypothesis, including positive and negative controls, and collected without technical issues. As data could be assigned to more than one category, the sum of percentages exceeded 100%. “Unfinished projects,” the largest source of unpublished data, were reported by 82% of respondents and represented ∼50% of their unpublished data. “Orphan data” was the second most common category at 47%, followed by data producing “negative (null) results” at 34%. Write-in responses in the “other” category (19%) echoed issues related to unfinished projects, such as personnel changes, lack of time to write, or difficulty publishing (Figure S1A). The age of unpublished data are shown in Figure S1B.

To prepare for the survey creation, we informally interviewed 13 scientists regarding their unused resources. These discussions revealed wide differences of opinion regarding whether negative results were “publishable” and prompted us to ask respondents to remain unbiased as to their data’s projected value or impact. To quantify varying opinions regarding the publishing of negative results, we presented respondents with various statements and ascertained the degree to which they agreed, disagreed, or held no opinion (Figure 1C). The strongest opinions were that publishing of negative results was important (agreed-strongly agreed; mean ± s.e.m 1.2 ± 0.5), but that publishing negative results in respected journals was difficult (agreed-strongly agreed; 1.25 ± 0.56).

Next, we sought to quantify the amount of unpublished data respondents had accumulated. To proceed, we defined a “unit” of unpublished data as being “sufficient to create a single table or graph.” We took this approach so that respondents could answer based on conventions within their field and area of research. In addition, this estimate would capture subsets of “orphan data” that may have been excluded from a larger set of data used for publication.

In keeping with this definition, most respondents estimated that they possessed 10–24 units of unpublished data, with over 60% possessing between 5 and 50 units (Figure 1D). Respondents were then asked to estimate the typical cost to produce one unit of data, including the cost of materials, supplies, services, etc. but excluding the salary costs of full-time laboratory personnel (Figure 1E). Most estimates (60.8%) fell between $100 and $2,999 but 10% of respondents estimated that the average unit of data cost them more than $10,000.

The midpoints of these ranges were then multiplied (quantity × cost) to calculate the approximate estimated value of unpublished data for each researcher. These values were stratified by field (Figure 1F) and tier/sector (Figure 1G). Researchers in engineering and life sciences possessed the highest median value of unpublished data ($31k), followed by the physical sciences ($13k), and social sciences ($5k). Researchers in the government sector possessed the costliest data ($56k), followed by R1 ($28k), D/PU and M1-3 ($17k), and R2 ($13K) institutions.

While these numbers represent self-reported estimates for the costs of data collection and processing over a range of data types, the findings demonstrate that unpublished data may represent a significant potential loss of resource investment and that systemic issues such as personnel turnover, time constraints, and perceived publication biases contribute to deficits in their utilization.

### Publication efficiency and publication pressure

**Figure 2:**
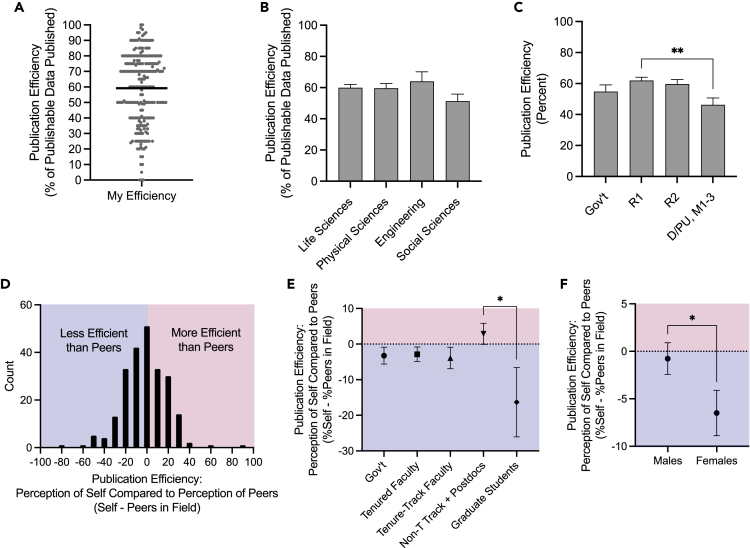
Publication efficiency estimates of self and peers Participants were asked to imagine a theoretical scenario in which “100% efficiency = publishing 100% of all publishable data in peer-reviewed journals,” in keeping with previous definitions of “publishable data”. (A) Self-reported estimates of publication efficiency from all respondents, with mean indicated by black line. (B) Comparison of mean publication efficiency by field and (C) sector/tier. Mean and s.e.m shown. ∗∗p < 0.01, One-way ANOVA with Tukey’ multiple comparisons. Participants were then asked to estimate the publication efficiency of the average research laboratory in their field, noted as “peers in field.” (D) Histogram of differences between self and peers. (E and F) Differences stratified by position (E) and (F), gender. Mean and s.e.m shown. ∗p < 0.05, One-way ANOVA with Tukey’s multiple comparisons (e) and unpaired t-test (f). *n* = 230–240.

To gain further insights into how researchers viewed their own publishing activity, we asked respondents to imagine their publication efficiency, a theoretical scenario in which being 100% efficient means publishing 100% of all “publishable” data in peer-reviewed journals. These were compared to self-reported estimates of publication pressure (Figures S2A–S2C). Estimates of publication efficiency were normally distributed with a mean, s.d. of 59.18 ± 23.05% (Figure 2A). Publication efficiency did not significantly differ between researchers in different fields (Figure 2B) but was lower at D/PU and M1-3 academic institutions (Figure 2C) where perceived publication pressure was also reduced (Figure S2B). Among individual researchers, there was no significant relationship between publication pressure and publication efficiency (p = 0.70) nor the number of estimated unpublished data units (p = 0.83). There was an association between the number of unpublished data units and publication efficiency (*r*_*s*_ = −0.22, p < 0.01).

We then asked respondents to estimate the publication efficiency of the average research laboratory in their field. Differences in the perception of efficiency between self and peers are shown in Figure 2D. On average, individuals felt that they were as efficient or slightly less efficient than their peers (mean, −3.19%; median, 0%); however, greater differences emerged when respondents were stratified by position (Figure 2E) and gender (Figure 2F). While the number of estimated unpublished data units were identical between males and females (mean, median of 10–24 units of data), females viewed themselves as being significantly less efficient (−6.50% versus −0.76%; p < 0.05). Together, these data grant additional psychological and sociological insights into the research and publication process.

### Unused samples

Next, we asked respondents about their unused samples. In order to include as many research fields as possible, we broadly defined an unused samples as “samples/specimens that are produced in excess, left-over from experiments or collections, or so easily generated that the laboratory would be willing to share them with other respected collaborators” and exclude “samples that the laboratory would be unwilling to share or are not suitable for use in publication due to insufficient rigor and/or reproducibility standards or technical/experimental issues.” Given the wide range of fields we included in the survey, we allowed each researcher to decide what constituted an “unused” “sharable” sample within their specialty.

**Figure 3:**
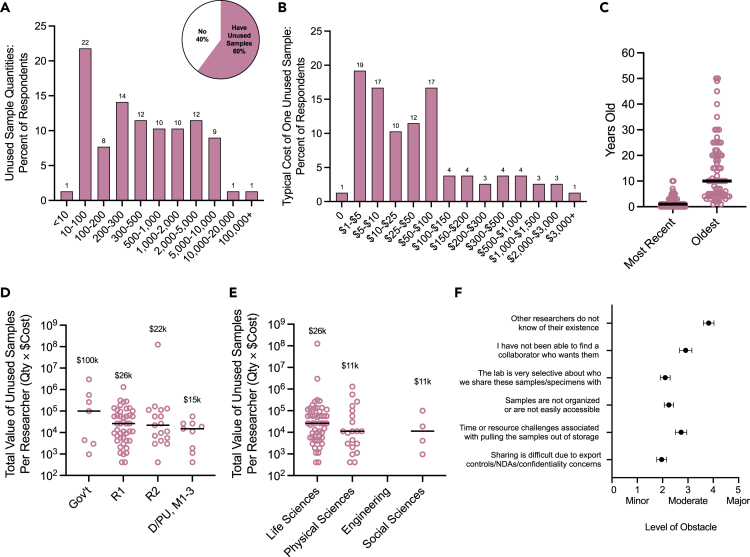
Self-reported estimates of the quantity and cost of unused samples Unused or left-over samples were defined as “samples/specimens that are produced in excess, left-over from experiments or collections, or so easily generated that the laboratory would be willing to share them with other respected collaborators.” This excludes samples that the laboratory would be unwilling to share or are not suitable for use in publication due to insufficient rigor and/or reproducibility standards or technical/experimental issues. Of the 53% of respondents whose research entails sample or specimen collection, 60% indicated that they had unused samples that met this definition. These individuals then answered questions about the estimated quantity and cost of their unused samples. (A) Assuming 1 specimen, tube, sample, aliquot, etc. = 1 Unit, percent of respondents with indicated unused sample quantities. (B) Percent of respondents indicating the average estimated cost to generate one sample (excluding employee time cost). (C) Age of samples. (D and E) Approximate estimated value of unused samples per researcher (avg quantity × avg cost) stratified by field (D) and (E), sector/tier. Median indicated by lines and annotations. (F) Rating of obstacles preventing the sharing of unused samples with others. Mean and s.e.m. shown. *n* = 78–250.

A total of 53% of survey respondents collected samples or specimens as part of their research. Sixty percent of these individuals indicated that they had material meeting the definition of unused samples/specimens, with wide variation seen among respondents. Respondents were also asked to provide examples of their unused samples, which included, “serum and tissue from infected animals,” “whole animals; frozen tissues,” “frozen fruit flies from a selection experiment,” “embedded mouse brain tissue,” etc. (Data S3). A range of 10–100 samples was the most common answer (22%), but the median answer corresponded to 300–500 samples (Figure 3A). Respondents were then asked to estimate the average cost required to generate one sample, including the cost of materials, reagents, services, maintenance fees, disposables, etc. but excluding salary costs for full-time laboratory employees (Figure 3B). Seventy-five percent of respondents estimated this cost to be between $1 and $100 per sample. The age of samples reported ranged from 0 to 50 years (Figure 3C), implying gradual accumulation of unused samples over time.

For each researcher, the midpoints for the ranges they selected were multiplied (quantity × cost) to calculate the approximate estimated value of unused samples. These values were stratified by field (Figure 3D) and tier/sector (Figure 3E). Researchers in the life sciences possessed the costliest unused samples (median of $26k), followed by the physical and social sciences ($11k). None of the engineering and technology respondents possessed unused samples that met the specified criteria (Figure 3 legend). In terms of sector and tier, government researchers had the most expensive unused samples ($100k), followed by R1 researchers ($26k), R2 ($22k), and D/PU and M1-3 ($15k).

In addition, respondents identified obstacles that interfered with their ability to share their samples with potential collaborators (Figure 3F). These researchers reported that others not knowing of the samples’ existence or not being able to find collaborators as the largest obstacles compared to accessibility, selectivity, resource challenges, or confidentiality.

### Total unused resources

**Figure 4:**
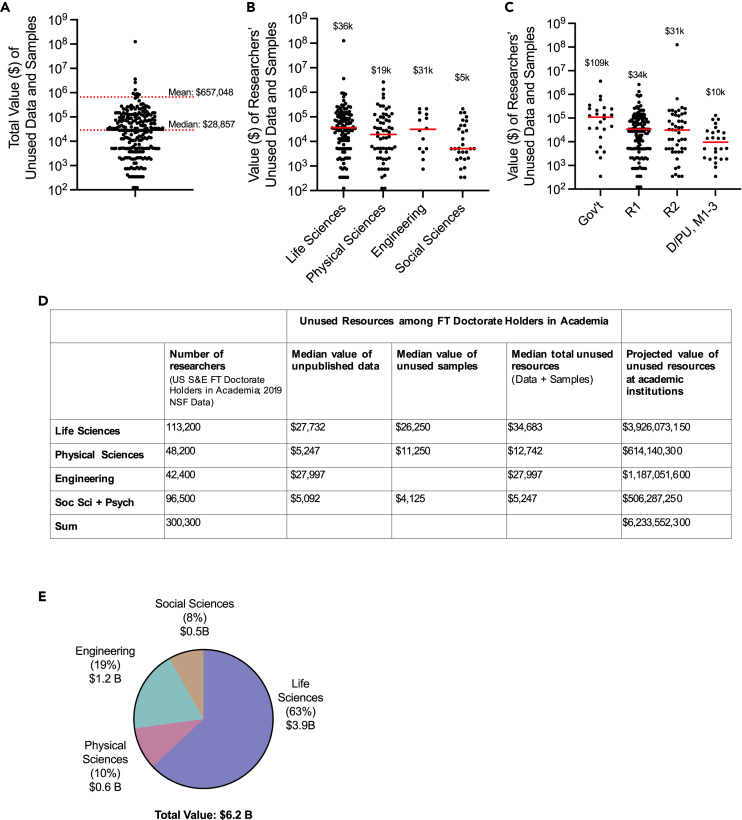
Estimate of the unused laboratory resource economy at US academic institutions For each respondent, the self-reported estimates of unused data and samples were added together to calculate the total estimated value of their unused resources per researcher (A). These values were then stratified by field (B) and sector/tier (C). Medians are indicated by the red lines and annotations. (D) To estimate the value of unused resources at US academic institutions, the median total value of unused resources (data + samples) was re-calculated for respondents who were full time (FT) doctorate holders in academia only. This value was then multiplied by corresponding NSF science and engineering (S&E) headcounts in each field.^25^ (E) Size estimate of the unused resource economy at US academic institutions.

To estimate the total cost of unused resources per investigator, the self-reported estimated costs of unpublished data and unused samples were summed for each researcher (Figure 4A). Across all researchers, the median value of total unused resources was $28,857. However, the possession of very costly assets among some researchers skewed the mean to $657,048. Researchers in the life sciences ($36k) and engineering ($31k) had the most expensive median unused resources compared to the physical ($19k) and social sciences ($5k). The median value of unused resources for government researchers ($109k) was approximately three times as high at R1 institutions ($34k), followed by R2 ($31k) and D/Pu-M1-3 institutions ($10k).

These data were then used to estimate the total value of unused resources at U.S. academic institutions. We recalculated the median total unused resource value for full-time doctorate holders in academia only and then multiplied this number by corresponding NSF headcounts^25^ across research fields (Figure 4D). The resulting calculations projected a total of $6.2 billion in unused laboratory assets, the bulk of which may reside within the life sciences (65%, $3.9b; Figure 4E). Engineering researchers were estimated to hold 20% of unused assets, $1.2b, followed by the physical sciences (10%, $0.6b), and social sciences (8%, $0.5b). Although government researchers were included in our survey, they were excluded from this computation as similarly stratified headcount data were not available for this group.

## Discussion

Economists define “stranded assets” as “assets that have suffered from unanticipated or premature write-downs, devaluation, or conversion to liabilities.”^26^^,^^27^ While this term is often used in the context of devalued real estate or inaccessible fossil fuels, it is highly applicable to unpublished data and unused samples among government and academic researchers. In analogous terms, the research process distills investments of time and money into data, the value of which is scientific knowledge and advances to meet societal needs. In some research fields, the generation or acquisition of samples is a vital, resource-intensive precursor to obtaining and using data. In addition, the publications that originate from scientific activity provide value in the form of career advancement, ability to obtain future grant funding, and societal impact. However, when data remain unpublished and samples are left unused, the value of these investments becomes “stranded” in a state of stored potential. In cases where these materials are time-sensitive or their existence remains unknown, their value may be lost completely.

Our data suggest that the average researcher possesses about $29,000 in stranded assets, which means that the average R1 institution has millions of dollars in unused assets and, across US academic institutions, a grand total of $6.2 billion may be at stake. It is important to place these findings in temporal context and emphasize that this is a cumulative snapshot of unused resources. The rate of aggregation is currently unclear, as some respondents’ answers may represent an entire career’s worth of accumulated unused resources, while junior investigators may possess only a few years’ worth. While this figure does encompass an enormous amount of resources, it represents a fraction of the total academic R&D spending in a given year—about 7% of the $90 billion spent at universities in 2021.^28^ Moreover, it should again be emphasized that is not an estimate of “waste” but instead the amount of stranded assets that are in danger of becoming waste.

Here, we extrapolated the approximate value of unused resources in academia; however, the total unused laboratory resource economy in the U.S. is likely orders of magnitude larger when considering inputs from the government sector and private industry. Others have given much larger estimates. In their *Lancet* paper on research waste, Glasziou and Chalmers (2009) surmised that the percent loss attributed to non-publication alone (if data passed muster in other areas) would be ∼25%. Given NIH’s $45 billion annual budget,^29^ this would translate to about ∼$11 billion each year for biomedical research alone.

Our findings suggest that data are often not withheld by choice, as only 8% of respondents indicated that they possessed data they did not want to publish. Instead, the evidence indicates that systemic issues and publication bottlenecks hamper the efficient conversion of data into peer-reviewed publications. During the manuscript preparation process, carving out a cohesive publishable unit from results often leads to data being “orphaned” because some datasets are not sufficiently robust to be published on their own or may be regarded as tangential. Indeed, 47% percent of respondents possessed unpublished data that meet this description. Over a third possessed unpublished negative (null) results and felt strongly that these data are undervalued by peers and publishers. Finally, unfinished projects were by far the primary source of unpublished data with personnel turnover and a lack of time identified as major culprits. These results echo findings from other studies where lack of time, directionality of findings, and stalling in the preparation/review process have all been identified as causes of unpublished data.^7^^,^^12^^,^^30^ With respect to unused samples, it appears that the opportunity to convert these stranded assets into usable resources via collaboration is stymied primarily by a logistical constraint: widespread knowledge of their availability.

Despite the obstacles faced by researchers in publishing, most feel their performance is commensurate with their peers. However, further stratification reveals that women tend to view themselves much more critically than men despite reporting identical quantities of unpublished data; a discrepancy that many would ascribe to “imposter syndrome.”^31^^,^^32^^,^^33^^,^^34^ Although the literature is divided over the effects of gender on imposter syndrome, this finding supports previous reports that females experience higher rates than men.^34^ Also of note is that the amount of publication pressure individuals experienced had no bearing on their perceived publication efficiency nor the self-reported amount of unpublished data. Together these data provide a unique view the intersection of scientific productivity with factors such as stress, systemic issues, resource limitations, and self-image.

While it is not realistic to expect that all the challenges identified here can be completely overcome, there is hope that solutions can be identified. In the past 10 years, much attention has been given to shifting the publishing framework toward open science as a means of facilitating data sharing. As these practices become more mainstream, they represent an essential way to reduce waste in the form of unpublished data. Moreover, they may represent the one of the few options to overcome publication bias against negative or null results.

While continued efforts in this area are valuable, our findings argue that tackling unfinished projects would also be fruitful, as they are a predominant source of unpublished data. In regard to unfinished projects, perhaps the greatest challenge is the fact that career development timelines and financial pressures are often not compatible with the pace and compensation structure of research. In academia, those directly involved with data generation (typically students and early career researchers) often leave their positions to acquire better salaries and to advance their careers, leaving behind unfinished projects. Indeed, our data provide actionable evidence and the financial impetus that decision-makers could use to justify policy changes targeting these problems. For example, requirements for better research planning can be put in place, such as transition plans for personnel changes and institutional incentives for completing projects and publishing manuscripts and/or raw data. One survey respondent suggested an “institutional requirement for delivering all unpublished data with details required for publication and an agreement for authorship before departure from the lab.” As the financial loss from unfinished projects is significant, implementing incentives to conclude experiments, publish results, and mentor replacement staff could also be justified.

There is also an important lesson to be learned from conventional “stranded assets” in economics: the way to make use of them is to repurpose them. Similarly, unpublished data and unused samples can be mobilized through new applications and collaborative efforts. Another respondent commented “sometimes we don’t see value in data … but others might find it very valuable” and suggested the need for collaborative tools and exchanges to helps scientists “make mutual agreements for analysis/publication.” Such an approach could help make use of hard-to-publish negative results, reproducibility studies, pilot data, orphan data, or ancillary findings and would help overcome the central challenge of unused samples: knowledge of their availability.

### Limitations of the study

These findings are based on the parameters and definitions set forth in the survey, which could under- or over-estimate the actual pool of unused resources in several respects. First, data that researchers anticipate will take longer than one year to submit to a peer-reviewed journal are counted as “unpublished.” This cut-off was established because many papers can stall in the manuscript preparation and the likelihood of publication tends to declines over time^7^^,^^12^; however, it is not uncommon for some completed projects to take multiple years to be submitted, therefore some data could have been prematurely counted as “unpublished.” Moreover, we did not count stand-alone submissions in data repositories or pre-prints, although many such data would have been included in our estimates as they are often posted ahead of or in tandem with peer-review publications. Alternatively, the survey could have underestimated resources in that it did not quantify data that still need work in order to become “publishable.” Finally, we did not include the cost of employee time and effort in the value of data and samples, as we felt this would be too difficult to disentangle within the confines of this survey. This is arguably the most resource-intensive aspect of data and sample generation, which leads us to hypothesize that findings are overall likely underestimated.

Another challenge of this survey was that the generalizability of the questions made it difficult to capture field or institution-specific idiosyncrasies. This is particularly challenging because some “units” of data may be large and complex, such as cohort or population data, and some may be small, such as data from a simple experiment.

As with any survey, concerns regarding response bias must be acknowledged. At 5.5%, our response rate was on the low side, but not unexpected for a “cold” email survey and the characteristics of population being surveyed (i.e. busy professionals).^35^^,^^36^^,^^37^^,^^38^ However, lower response rates are not necessarily correlated with response bias^39^^,^^40^ and previous studies have demonstrated that response rates of 5% in similar respondent populations produce reliable survey results provided a sampling frame of at least 500 individuals.^41^ In support of this interpretation, our publication efficiency statistics of ∼60% align closely with previously reported publication rates of 58%,^12^ ∼45%,^3^ >50%.^4^

## STAR★Methods

### Key resources table

**Table:** 

REAGENT or RESOURCE	SOURCE	IDENTIFIER
**Deposited data**		

Survey Data	Mendeley Data	https://doi.org/10.17632/6zxhcdsf7x.1

### Resource availability

#### Lead contact

Further information and requests for resources and reagents should be directed to and will be fulfilled by the lead contact, Dr. Emma Bowers (emma.ciel@gmail.com).

#### Materials availability

This study did not generate new unique reagents.

### Experimental model and study participant details

In compliance with the institutional policy for human subjects research, the survey protocol was reviewed by the University of Arizona Institutional Review Board and deemed exempt from full review. The selection process is described in method details and respondent characteristics are included in Table 2. We did not collect information on ancestry, race, or ethnicity.

### Method details

#### Survey creation

Prior to preparing the survey we informally interviewed 13 academic scientists about their unused data and samples, which provided preliminary information as to the scope of the problem and underlying issues. Using this information, survey questions were developed to assess the quantity and value of unused data and samples and the reasons for their disuse.

#### Selection process and deployment

US academic institutions were stratified by Carnegie Classification and a random number generator was used to select those to be included in the study. We selected 22 R1, 15 R2, 15 D/Pu, and 15 M1-3 institutions. For each institution, we sought individuals from the biomedical sciences, biological sciences, medicine, earth sciences, engineering, chemistry, physics/astronomy, and social sciences (psychology, sociology, anthropology). In addition to comprising the bulk of R&D, we focused on the aforementioned fields because they are the most likely to utilize tangible data and samples and capable of being assessed by our survey instrument.

Within each subject area, we assembled contact lists from publicly available online institutional directories. Contacts consisted primarily of tenured and tenure-track faculty (65-80%, depending on the institution type). Research staff, post-docs, and graduate students were also included if their contact information was available.

Similarly, we assembled a list of government research laboratories and institutes and randomly selected 30 for study inclusion; however, contact information for many government agencies was limited. We did not include researchers in industry given the differences in organizational structure, staffing, publication pressure, and funding mechanisms.

In total, 9,149 contacts from academia and 1,057 contacts from government researchers were collected. In addition, 176 invitations were sent out on LinkedIn to increase response rates. Informed consent was obtained on the first page of the survey. All respondents indicated that they were at least 18 years old, worked in a U.S. based laboratory, and were directly involved in research. The survey was deployed from May 30, 2022 to September 2, 2022 using SurveyMonkey software, which also sent invitation and reminder emails. Responses were collected anonymously.

### Quantification and statistical analysis

Statistical analysis was conducted using SPSS Statistics and Graph Pad Prism. Results were considered to be significant at *P*<0.05. Samples sizes ranged from 78-301 and are indicated in the legend of each figure. Unpublished data and unused samples were presented as percent of respondents, median, and mean, where indicated. Multiple comparisons were made using a one-way ANOVA with Tukey’s multiple comparisons. Single comparisons were made using an unpaired t-test. To calculate the value of unused resources at academic institutions, NSF headcounts of full-time doctorate holders in academia stratified by field were obtained from https://ncses.nsf.gov/pubs/nsb20212/table/SLBR-21. These numbers were multiplied by the median estimated value of unused total resources for US doctoral holders in academia (obtained from the survey results; excluded government researchers, students, and lab managers). We did not include estimates for the fields of mathematics and statistics and computer science.

## Data Availability

• The survey data been deposited at Mendeley Data and is publicly available as of the date of publication. The DOI is listed in the key resources table. • This paper does not report original code. • Any additional information required to reanalyze the data reported in this paper is available from the lead contact upon request.

## Supplemental information

Document S1. Figures S1 and S2 and Data S2 and S3

Data S1. Copy of survey questions, as described in STAR Methods
