# Supplementary material for: Scope and financial impact of unpublished data and unused samples among U.S. academic and government researchers
Source: iScience. 2023 Jun 19;26(7):107166. doi: 10.1016/j.isci.2023.107166 (PMC10359936; doi:10.1016/j.isci.2023.107166)
Supplement: Document S1. Figures S1 and S2 and Data S2 and S3 [file mmc1.pdf]

iScience, Volume *26*

Supplemental information

Scope and financial impact of unpublished data and unused samples among U.S. academic and government researchers

Emma C. Bowers, Jimena Stephenson, Melissa Furlong, and Kenneth S. Ramos

Data S2. Supplement to Figure 1: Respondents' self-reported examples of their unpublished data

An allozyme-based study long ago superseded by sequence data; microarray-based gene expression data;
Analysis of a subsume of the parameter space of string landscape.
Analysis of noise effects in 3D reconstructed images; kinetic theory calculations
Analysis of terpenes in wastewater
Animal behavior data; Sequence data
Animal behavior studies
Animal community surveys; Behavior study of an endangered species; Stable isotope analyses
Animal studies of immune function, protien interaction studies, gene expression studies
Arctic planetary analog studies; Planetary mapping
Artifact technological data; artifact functional or chronological data; archaeological site assemblage or chronological data
Astronomical observations taken at Kitt Peak observatory
Attitudes and perceptions on local environmental issues
Basic science research
Behavioral data, community analyses
Biochemical data from cultured cells and animal brains
Biochemical neuropharmacology of GPCR signaling.
Biophysical protein characterization; Single molecule trap measurements; Computational simulation results
Brain imaging analyses
Cancer gene expression analyses
Cardiovascular effects of acetaminophen; Pathological effects of high vs. low dose atorvastatin.
Cell culture tweeks that may be beneficial.   Incomplete Sample screenings   One off experiment
Cell morphology data; respiratory physiology data
Cell respirometry; measures of enzymes relating to acid-base equilibrium
Characteristics of individuals who engage in cyberbullying behaviors; Descriptive study of individuals enrolled in a medication review program; Evaluation of a hospitalization prevention program focused on individuals living with dementia
Characterization of mutant phenotypes; Transcriptome analysis.
Chemical reactor performance results
Chemical studies
Chemical synthetic methodology development applications
Classroom data on student understanding of Nature of Science; Student surveys on using technology to track understanding of student learning objectives in real time
Clinical research projects
College student survey data; Court case data
Comparison of treatments to control invasive common reed Phragmites in a saltmarsh
Computational model results
Computational models of enzyme mechanisms; Computational models of organometallic complexes.
Confocal tilescan images from giant vesike.
Consult service data; Project ECHO data
CT and X-Ray scans of turtles for systematics; Turtle genetics; Reproduction data
CT scans and biomechanical simulation results
Culturally Responsive approaches to teaching American Indian Alaska Native Students; Culturally Emotional Learning; Consultation with American Indian/Alaska Native Clients
Demographic data on fish populations; Effects of invasive quagga mussels on fish populations; Otolith microchemistry of yelloweye rockfish
Development of advanced conductors
Diary data, observational data (recordings), survey data
Diet and health outcomes
Dietary intervention studies

Disinfectant toxicity
Distributions of particulate matter of unusual size fractions; Air concentrations of trace species below public health relevance; Modeling results of air cleaners performance
Diversity and prevlance of pathogens; Pathogen identification
DNA sequence data; ecological interactions; chemical analysis
Drug delivery using engineered nanoparticles
Dynamic kinetic resolution of 1-arylalllyl alcohols kinetic resolution of 1-arylpropargyl alcohols
Mechanism of smoking toxicity, E.g. Animal experiments not confirming hypothesis, E.g. partially solved questions
Eco-physiology of macroalgae under warming temperature; descriptive community and monitoring data
Ecoenzymes and amplicon sequencing of biological control communities in soil associated with soilborne disease; Temperature effects on Phytophthora root rot of tomato
Effect of novel protein modulators on triglyceride metabolism
Effects of nutrition in brain function
Effects of SARS-CoV-2 on GI tract; Effect of opioids on intestinal barrier function
Emotion regulation data
Environmental monitoring using a non-traditional methodology
Enzyme distribution; mechanism of toxicity; enzyme purification
Ethnographic fieldwork data
Evaporation rates of liquid explosives; Infrared spectra of electrical tapes
Evoking jealousy; Self expansion
Experimental results: spent blast abrasives reuse, impact of longterm flooding on asphalt materail, shear and pullout of connectors in sandwich laminate test results
Fabrication process development data
Farm management practices survey data; Qualitative interview data; Document analysis and archival analysis data
Features that create perceptions of obligations; The problem with treating distributions as reflective of persons in the study of social psychology; Reevaluating how unexpected events are represented in theory-of-mind regions; How conflicted minds cause events
Field studies from multiple years and locations; Undergrad student projects; Bench top trials
Fish assemblage data
Flow Cytometry data from clinical samples; Biomarker screening from cell lines
Fossil collections
Gene expression
Gene expression data and signaling data during stress in cells
Gene expression data from cultured hepatocytes
Gene expression data from human liver, viral infection data from cultured cells
Gene expression data from LCLs without exciting results; Correlations between genetic diversity and fitness; Whole genome gene expression from sparrows
Gene expression data from primary human and mouse T cells. Human intestinal organoids stained with Hoechst and Ki67. Proteome profiler array data of conditioned media from human placental stromal cells.
Gene expression data from seasonally breeding songbirds.
Gene expression data; Data generated during standardization.
Gene expression in T cells; Protein-protein interactions in activated T cells
Gene expression; behavior; neuroanatomy
Gene overexpression in cell lines
Gene regulation that impacts cancer cell aggressiveness
Genetic replication analyses; Attitudes to specialty care; Allergies in multiple sclerosis; Early childhood exposures in autoimmune demyelinating disorders; Experiences of stigma in multiple sclerosis; Genomewide association study of neuromyelitis optica spectrum disorder
Geochemical analyses and geologic mapping
Geochemical results and paleoecological tallies
GPS tracking data; Physiological data

Granzymes have no effect on recombinant perforin; CD107A externalization is a bad marker for lymphocyte-mediated killing; C reactive protein in chronic fatigue syndrome patients sera; NK phenotypes in 200 normal donors
Greenhouse gas fluxes
Human biomechanics
Human neurocognitive development
I wrote a program, I did not published yet. I did some insightful graphs for a class, but it's not enough to be published.
Identified mouse that suppresses tumor formation, never mapped locus   Have mouse lines that have been partially characterized
Image data sets (multiple Gb per sample). Often only
Imaging studies on human brain tissue mouse brain studies analysis of enzyme expression in heart
Immunogenomics; antiviral compounds
Immunohistochemistry and gene expression from mouse model
Immunology data on elk   hamster leptospirosis studies- several bactericidal studies
Impact of COVID 19 on families in Southeastern Louisiana
Inelastic neutron scattering data
Infant looking time data, senior theses for students who left,
Infection data from wildlife individuals
Infiltration measurements; unfinished lab characterizations; testing of new methods that do not always work out
Insect genome assemblies; gene expression studies from intoxicated insects; gene expression studies from phosphine-treated insects; RNAi
Isotope, solute, and field-parameter (pe, DO, pH, temperature, specific conductance) data from water samples.
Job pursuit motivations; hiring process attrition
Kinetic data from recombinant enzymes and their mutants
Confocal microscopy digital image data
Language awareness data; territoriality & geneaology studies
Lipidomics analysis from fruit fly heads; lipid staining in fruit fly eyes
Literature review; Geochronology data using novel lab methodology
Lithogeochemical analyses, mineral isotopic analyses, high resolution X-ray computed tomography data and models
Locations and info of galaxies in a sky survey
Long term effects of quarantine due to Ebola outbreak
Magnetic resonance spectroscopy measurements of the brain in a variety of diseases.
Many many cyclic voltammetry curves that are inconsistent for one reason or another
Maternal ratings of child temperament
MDS scaling data representing changing mental representations of spoken vowels; Psychometric validation of a video comprehension measure; Small N (more than 10 per cell but less than 100 overall) research studies
Mental health impacts of long-distance hiking
Microbial community analysis data from several years in soil. Functional gene (nrfA) analysis data from soil as a function of depth.   Analysis of N2O consumption from soil in response to diurnal temperature fluctuation.
Microfossil analyses of lacustrine deposits from western North America and Hawai'i (modern and fossil)
Microprobe analyses of mineral suites; X-Ray tomography of metamorphic rocks; Geologic maps; Kinetic models of metamorphic reaction mechanisms
Microsatellite genotypes, stable isotope data for food web studies, invertebrate community data
Mineral and rock compositions; models; gravity measurements; stratigraphic measurements
Molecular Dynamics Analises
Mostly telescope observations that I haven't gotten around to analyzing yet.
Mycorrhizal fungi community data

Natural history observations. Documentation of behaviors. Simulation/modeling results. Analyses that could not be published immediately because of time constraints, and have become a bit obsolete.
Neurobehavioral or neuroimaging studies of children; Behavioral toxicology studies in animals
Neuroscience electrophysiology data without enough samples; Neuroscience behavioral data with incomplete analysis
Non protein vaccine trials in Rhesus Macaques
Numerous undergraduate theses; Data needing further analyses; Data collected to support some contractual work
Observational trial of coinfection in dairy cattle; Ongoing surveys still in progress (mostly tick)
Occupational biomechanics of power tools and gloved hands
Organic carbon measurements from a research cruise
Orphan data: behavioral neuroscience study; rejected study: pedagogy (psychology)
Papers from Kuwaiti students who interviewed their families about modernization and tradition in the 1970s.
Parkinson's disease data related to cognitive and emotional function
Particle size distribution and temperature profiles for crystallization of ibuprofen
Particular experimental conditions that can reveal things unrevealed in similar settings.
Patient opinion data; Practice demographics; Treatment results
Petrographic observations; Geochemical data
Petrologic, geochemical, and paleontologic data from Paleozoic and Mesozoic rocks in various areas of Alaska
Phylogenies, taxonomic revisions, and new species descriptions
Physiological measurements such as blood pressure, heart rate, etc under disease states in animal model
Pilot data when developing a new technique, or confusing data where the controls didn't work or we have other reservations about it
Plant physiology
Policy analysis
Polymer coated urea impacts on yield and environmental nitrogen losses; Enhanced efficiency phosphorus fertilizer impacts on yield and environmental phosphorus losses; Water conservation practices in agriculture; Water conservation practices in the urban environment
Polymer synthesis, resin characterization, adsorption data
Population and community ecology data; genetic, transcriptomic and genomic sequences
Power system test cases
Precipitation and soil modeling; invasive plant competition
Prediction of consumers quality of meat by HSI'
Protein and gene expression in smooth muscle
Protein methylation with PRMT enzyme; Comparing male and female autoantibody detection in diabetes; Detecting nitrated proteins on microarray
Public health survey data; Medical groups data; Neuro imaging data; Cognitive testing data
Quantitative genetic analyses histology
Radar measurements of tree height landslide observations
Radiology methods
Raw Geological Field data and measurements
Reaction mechanism data
Reaction mechanisms
Remediation data
Remote sensing analyses.   Gridded rainfall composite analyses.   Natural resource manager survey data.   Derived model products.
Results from genetic surveys of natural population
RNA seq analysis, genetic sequences from species not studied
RNA sequencing data; Phylogenetic analyses; Biochemical data

Role of attachment and other resilience related factors as a buffer of childhood victimization experiences. Pluralistic ignorance in environmental attitudes. Pluralistic ignorance in transgender attitudes. Pluralistic ignorance in COVID Related Health Behaviors.
Root turnover in response to climate change
Salmon behavior to dam operations
Secondary Electron Emission Yield on Novel Coatings; Simulation of radiation; Radiation-damage experimental results
Sediment transport to marshes during cold fronts in a microtidal setting
Sequencing data.
Simple clinical intervention study presented in 2 abstracts but manuscript never completed by student; Funded feasibility trial where aims were met with conclusion that project was not feasible
Small effects on gene expression
Small mammal capture recapture data, vegetation studies, reptile and amphibian samples, butterfly null results, ant pit fall samples, unpublished masters theses
Small results which do not fit in the bigger picture; some project stranded in development
Small-mammal trapping data
Social media posts; packet traffic; busroute usage
Soil microbiome data; protein expression data from leaves; plant physiological traits; bacterial growth curves; soil bacteria isolates
Solid-state behaviors and vibrational analysis of powdered pigments
Spatial and temporal occurrence of botulism outbreaks, results of simulation model of parasite competition, bird camera photographs categorized into presence/absence matrices through time
Spectroscopic data from Mossbauer and X-ray spectroscopies; Chemical data (aqueous species speciation and concentration); Study participant survey data
Stable isotope data
Stable isotopes of marine sediment
Strait of Gibraltar transport data; Coastal ocean model simulations
Structural member deterioration data; Structural behavior results from parametric analyses
Study of reading disability in 8-12 year-old children.
Supernovae from a robotic telescope; Un-reprocessed images from the same robotic telescope; Catalogs of galaxies that are in intermediate states of preparedness
Survey data on body image; Historical data on lynchings in the Midwest; nterview data on Black Lives Matter
Survey data; psychology experimental data; data from student projects
Synchrotron-based x-ray absorption spectra and fluorescence maps
Synthesis of new palladium complexes; reactivity data for palladium complexes; nitrene reaction results; nitroarene reduction chemistry; carboxylic acid reduction chemistry; proteomic data on neurotrophic responses
Synthetic methodology
Techniques of bayesian parameter estimation
The data which cannot be translated for real world problems. Not suitable for public health.
Trace element partitioning data, mainly in metal-silicate systems; Stable isotope fractionation data for metallic systems
Tracer studies in ecosystem and watershed science
Training evaluation data; Large longitudinal data set of pregnancy that has had some things published, but not all.
Transcriptome data; data on hybridization and introgression; data on reptile feeding preferences; field data on amphibians
Transcriptome sequences that haven't been analyzed; SNP genotypes with low amplification success; Morphometric data; Phylogeographic studies based on mtDNA only; Phylogenetic data with incomplete sampling; - Population genetic data that has yet to be analyzed
Transcriptomic studies, promoter activity studies, animal studies

Ultrasound images, preliminary studies to work out and optimize experimental design, data which needs more depth
Utilization of transitional care visits
Walrus carcass counts; Aerial waterfowl surveys in Lake Michigan
Water catchment data
Water level data sets without analysis and interpretation; data sets that have not been analyzed; student work that has not been written up
X-ray crystal structures of various metal complexes.
X-ray diffraction and electrical conductivity data from geophysics experiments
X-ray emission data from x-pinch
Zircon U-Pb data, zircon trace element data, mineral inclusion assemblage data, x-ray tomography images

Data S3. Supplement to Figure 3: Respondents' self-reported examples of their unused samples

Alaska fish frozen tissues; Otoliths from fish
Antibodies
Bat wing tissues; unanalyzed samples from infection studies.
Cell lines over expressing membrane proteins; Plasma from hospitalized patients and outpatient controls.
Chemicals
Cytotoxic granule extracts from NK lymphocytes
Digitalized bones of puma and leopard
DNA from ticks and from small mammals
DNA Samples for Endangered Turtles
Dried fecal samples
Dried plants, powdered (and chemically treated) bioapatite, collagen, fur and hair.
Dried sediment, leaves, or animal tissues; frozen leaf material
Dried soil and vegetation samples
Embedded mouse brain tissue exposed to hyperoxia; Fruit fly lines with transgenic human cDNA; Frozen fruit fly heads
Formalin-preserved fish samples
Fractionated plasma, cell lines
Frozen blood samples from various infectious diseases
Frozen cells, serum, plasma
Frozen DNA samples.
Frozen fruitflies from a selection experiment (from 2 actually); frozen fruitflies from an inbreeding depression study
Frozen leaf tissue for RNAseq; seeds; mapping populations
Frozen milk, teat skin and animal bedding samples
Frozen mouse placenta samples
Frozen soil samples
Frozen soil samples; frozen tissues samples, formalin fixed invertebrate specimens
Frozen tissue (rat/mouse brain sections), blood samples
Frozen tissue from animal brains
Frozen tissue samples; DNA samples
Frozen tissues from fish in natural and hatchery populations; Ethanol preserved tissues from fish; Leftover genomic DNA samples; Leftover genetic samples after DNA library prep for Illumina sequencing
Frozen tissues from rats following dietary intervention; plasma from intervention studies
Frozen tissues from wild populations of many species of turtle.
Frozen Tissues; Parafffin embedded tissues blocks that were formalin fixed
Frozen tissues; plasma; primary tumor samples
Herbarium plant specimens; microscope slides of leaf epidermis
Leaf tissue for DNA, leaf tissue for stable isotopes, seeds
Living iso-female lines of Drosophila flies, and preserved flies from natural collections
Marine organisms tissue collected across several inter-tidal habitats
Marine sediments
Melted glacial ice samples; rock samples containing mica"
Metal complexes synthesized but not yet studied
Mine tailings from Leadville, CO area; Asbestos samples from several locations around the world
Mouse frozen tissues collected in particular conditions.
Parkinson's disease neuropsychological data
Pitfall trap collections, videotapes
Plasma from whole blood
Pollen collection from flowers, pollen collection from bees, bee specimens
Preserved invertebrate community samples
Rat brain tissue from feeding and/or DREADDs studies

Rock and fossil samples; powders remaining from rock samples that could be used for geochemical analysis; Rock samples could be used for petrographic, geochemical and/or age analysis.
Rock and mineral samples
Rock and sediment samples
Rock samples
Rock samples
Rock samples
Rock samples; Mineral separates
Rock specimens from a variety of metamorphic terrains. All well located and described in terms of topographic map location and outcrop maps from field notes. Those collected since 1995 will have GPS coordinates.
Rock, mineral, and ore samples
Rocks, unconsolidated materials (mine tailings, soils), laboratory-generated materials
Sediment
Serum and tissue from infected animals
Soil samples
Soil samples
Soil samples; plant tissue samples
Surface water and groundwater samples
Textile fiber fragments
Ticks stored in ethanol
Tissue homogenates; cloned genes
Tissue, DNA extractions, seeds
Tissues from wild songbirds
Unprocessed herbarium and silica gel-dried leaves for DNA extraction
Used human serum diluted 1:100 in 5%milk/PBST"
Whole animals; frozen tissues

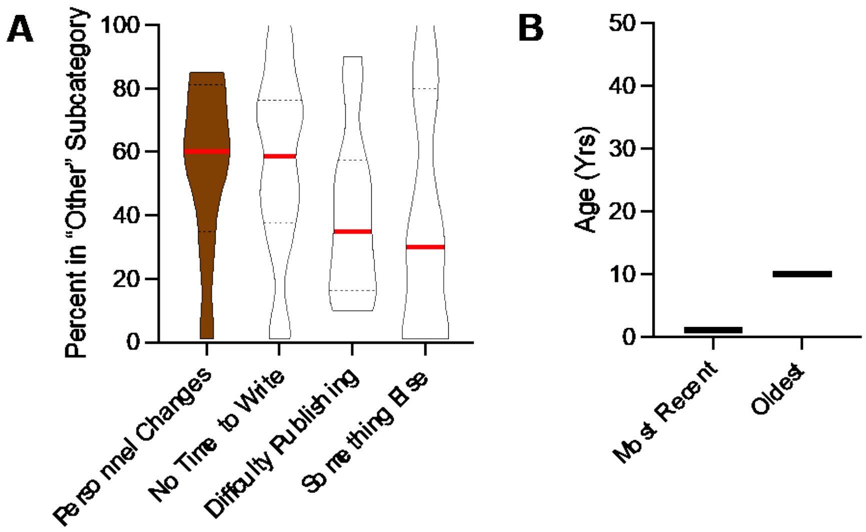

Figure S1. Supplement to Figure 1: Additional information regarding unpublished data. (*A*), Percent of data falling into subcategories of answers in "other" category from Figure 1B. (*B*), Age of respondents' most recent and oldest unpublished datasets.

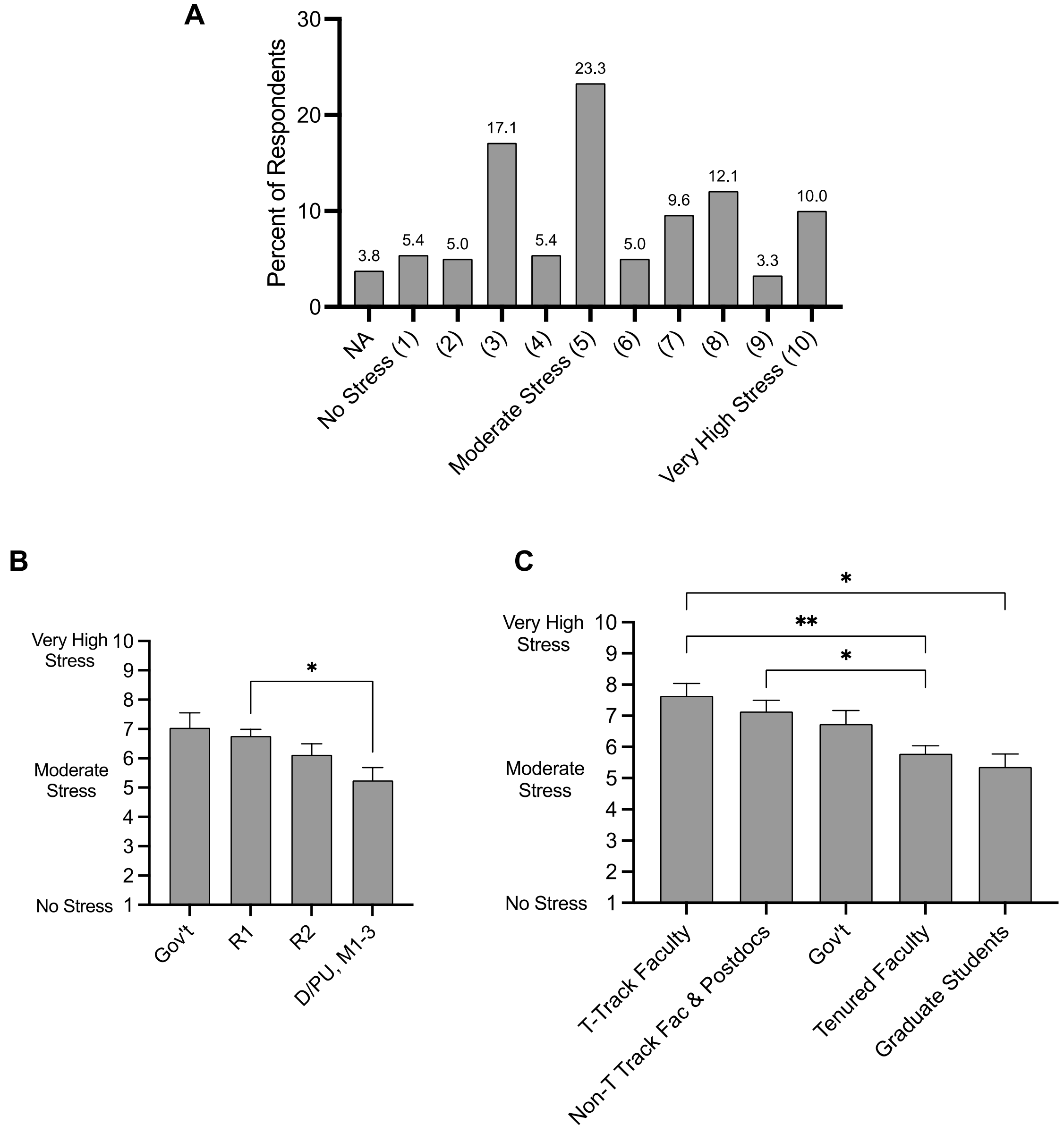

Figure S2. Supplement to Figure 2: Respondents' self-reported levels of publication pressure. Publication pressure was defined as the pressure to publish academic work in order to succeed in an academic or research career. (A) Publication pressure levels reported by respondents. (B) Publication pressure stratified by sector/tier and (C) position. (*B, C*), Mean and s.e.m. of responses; **P<0.01 One-way ANOVA with Tukey's multiple comparisons.
