# Supplementary material for: Scope and financial impact of unpublished data and unused samples among U.S. academic and government researchers
Source: iScience. 2023 Jun 19;26(7):107166. doi: 10.1016/j.isci.2023.107166 (PMC10359936; doi:10.1016/j.isci.2023.107166)
Supplement: Data S1. Copy of survey questions, as described in STAR Methods [file mmc2.pdf]

Cover Letter and Informed Consent

Dear Researcher:

You are invited to participate in a research study entitled "Unpublished data and unused samples: A survey of US researchers." This study is being conducted by researchers from the University of Arizona and the University of Arizona Center for Innovation.

The purpose of this study is to estimate the quantities of unpublished data and unused samples in research laboratories and to identify barriers to their use in future research. In this study, you will be asked to complete an electronic survey. Study participation is voluntary and you may withdraw your participation at any time. The survey should take less than 10 minutes to complete.

This survey has been approved by the Institutional Review Board of the University of Arizona. There are no risks associated with participating in this study. The survey collects no identifying information and all responses will be recorded anonymously.

While you will not experience any direct benefits from participation, information collected in this study will be used to identify ways to improve the efficiency of research.

If you have any questions, please contact eb.labsurvey@gmail.com. If you have any questions concerning your rights as a research participant, please contact the IRB of the University of Arizona (https://tinyurl.com/UAZIRB).

By proceeding with the survey, you are indicating your consent to participate in the study. Your consideration and participation is appreciated and key to the success of this study. Thank you.

Sincerely,

The Survey Team

Study criteria.

* 1. Please indicate whether you meet the following criteria:

- [ ] I am at least 18 years old
- [ ] I work at a United-States based research institution
- [ ] I am directly involved in research

Demographic Information

* 2. What gender do you identify as?

- ◯ Male
- ◯ Female
- ◯ Non-binary
- ◯ Transgender
- ◯ Prefer not to respond

3. What sector do you work in? Check all that apply.

- ☐ Academia
- ☐ Government
- ☐ Non-profit
- ☐ Industry

Institution Size

4. What best characterizes the size of your academic research institution?

The designations below are based on Carnegie Classifications. If you don't know the Carnegie Classification of your institution, you can quickly look it up here.

- ◯ **R1** Doctoral institution with "Very High Research Activity"
- ◯ **R2** Doctoral institution with "High Research Activity"
- ◯ **R3 or D/PU** Doctoral institution or professional university with "Moderate Research Activity"
- ◯ **M1-M3** Small, Medium, and Large institutions that award at least 50 master’s degrees per year
- ◯ Baccalaureate/Associate’s College
- ◯ Not sure / none of the above

Research Position and Field

* 5. What is your current research position? Check all that apply.

- [ ] Retired
- [ ] Physician (MD or DO)
- [ ] Veterinarian (DVM)
- [ ] Research Scientist or Principal Investigator (Government)
- [ ] Research Scientist or Principal Investigator (Industry)
- [ ] Tenured Faculty
- [ ] Tenure-Track Faculty
- [ ] Non-Tenure Track Faculty / Staff
- [ ] Postdoc
- [ ] Graduate Student: PhD
- [ ] Graduate Student: Master's
- [ ] Lab Manager
- [ ] Technician
- [ ] Undergraduate Student
- [ ] Other (please specify)

* 6. What is your primary field of research?

- ○ Biomedical Sciences
- ○ Medicine
- ○ Public Health or Epidemiology
- ○ Veterinary Medicine
- ○ Biological Sciences (Biology, Ecology, Evolution, Zoology, etc...)
- ○ Earth Sciences (Environmental Science, Geosciences, Hydrology, etc..)
- ○ Engineering and Technology
- ○ Chemistry
- ○ Physics/Astronomy
- ○ Social Sciences & Humanities
- ○ Computer Science

Unpublished Data

The follow section inquires about unpublished data.

Definitions:

Unpublished data:

- **Publishable data that have not been published in a peer-reviewed scientific journal**
- **Are not in articles currently under review**
- **Not a part of a publication to be submitted in the coming year.**

Publishable data: Data that are suitable for publication in a peer-reviewed scientific journal because they meet rigor and reproducibility standards in your field.
DO NOT exclude data from your estimates based on their projected value or impact (i.e. negative or inconclusive results)

Negative Results:. Publishable data in which there is a failure to reject the null hypothesis (i.e., an effect *doesn't* happen). For negative results to be publishable, the proper positive and negative controls must be included and there should be no technical or experimental issues.

* 7. We are trying to understand more about the reasons that data aren't published. Do you have any unpublished data that fall into the following categories?

- [ ] I don't have any unpublished data
- [ ] Negative results (Failure to reject the null hypothesis; Includes positive and negative controls; No technical issues)
- [ ] Unfinished projects
- [ ] Orphan Data (Data that doesn't "fit" well into the lab's other papers but does not constitute an entire publishable unit)
- [ ] Reproducibility studies
- [ ] Ancillary findings unrelated to lab's mission
- [ ] Data that I don't want to publish
- [ ] Other reasons (please describe)

Unpublished Data Continued

* 8. Using the selections from the previous question, estimate what percent of your unpublished data falls into each category. Some data may fall into more than one category.

I don't have any unpublished data

Negative results (Failure to reject the null hypothesis; Includes positive and negative controls; No technical issues)

Unfinished projects

Orphan Data (Data that doesn't "fit" well into the lab's other papers but does not constitute an entire publishable unit)

Reproducibility studies

Ancillary findings unrelated to lab's mission

Data that I don't want to publish

Other reasons (please describe)

* 9. Imagine a theoretical scenario in which:
100% Efficiency = Publishing 100% of all publishable data in peer-reviewed journals

Based on your impression, what is your estimate of the efficiency of the average research laboratory in your field?

0% 50% 100%

* 10. Estimate your personal percent efficiency.

0 50% 100%

* 11. Assume that 1 Unit of Data = Sufficient data to create a single graph/table.

Approximately how many units of unpublished data do you possess?

* 12. On average, how much money did it cost to produce one unit of data? Include the cost of materials, supplies, services, etc. You can average across types of data or types of experiments. Do not include the time cost of full time laboratory employees.

13. Approximately how long ago (in years) were your unpublished datasets collected?

- Answer in whole numbers (no decimals accepted).
- For data less than a year old, answer "0".

Most Recent (number of years)

Oldest Data Set (number of years)

Samples and Specimens

* 14. Does your research involve sample or specimen collection?

- ◯ Yes
- ◯ No

Unused Samples and Specimens

Definitions

<u>Unused or left-over samples:</u> Samples that are produced in excess, left-over from experiments or collections, or so easily generated that the laboratory would be <u>willing to share them</u> with other respected collaborators.

DO NOT include

- **Precious samples that the laboratory would be unwilling to share**
- **Samples/specimens that are not suitable for use in publication due to insufficient rigor or reproducibility standards or technical/experimental issues.**

* 15. Based on the definitions above, does your lab possess unused or left-over samples?

- ◯ Yes
- ◯ No

Questions about Extra Samples and Specimens

* 16. Approximately how many unused samples/specimens do you have in your laboratory? Assume 1 specimen, tube, sample, aliquot, etc. = 1 Unit

17. Approximately how long ago (in years) were your unused samples collected or generated?

- Answer in whole numbers (no decimals accepted).
- For samples less than a year old, answer "0".

Most Recent (number of years)

Oldest (number of years)

* 18. Approximately how much does it cost to generate <u>one</u> of these samples? You can generalize across sample types.

Consider cost of: materials, reagents, paid services, maintenance fees, disposables, etc. Do not include the time cost of full time laboratory employees.

* 19. Do any of the following obstacles prevent you from sharing your extra samples or specimens with collaborators? If so, please rank how much of a challenge each obstacle presents. If there are additional obstacles, please enter them under "other."

	Not Applicable	Not a challenge 0	Minor Obstacle 1	2	Moderate Obstacle 3	4	Major Obstacle 5
Other researchers do not know of their existence	○	○	○	○	○	○	○
I have not been able to find a collaborator who wants them	○	○	○	○	○	○	○
The lab is very selective about who we share these samples/specimens with	○	○	○	○	○	○	○
Samples are not organized or are not easily accessible	○	○	○	○	○	○	○
Time or resource challenges associated with pulling the samples out of storage	○	○	○	○	○	○	○
Sharing is difficult due to export controls/NDAs/confidentiality concerns	○	○	○	○	○	○	○

Other (Please specify obstacle and rank)

The Publication Process

* 20. Publication pressure can be defined as the pressure to publish academic work in order to succeed in an academic or research career.

How much stress from publication pressure are you currently experiencing?

* 21. Negative results indicate an effect *doesn't* happen, in other words, there is a failure to reject the null hypothesis.

Consider only *publishable* negative results in which the proper positive and negative controls were included and there were no technical or experimental issues.

How much do you agree or disagree with the following statements:

	Strongly Disagree	Disagree	No Opinion or Not Sure	Agree	Strongly Agree
In general, it is important to publish negative results	○	○	○	○	○
Negative results are an experimental dead-end and not something that I am interested in publishing	○	○	○	○	○
The negative results from my lab are valuable and I am interested in publishing them	○	○	○	○	○
It difficult to publish negative results in respected journals	○	○	○	○	○
I have not had any challenges publishing my negative results in respected journals	○	○	○	○	○
A paper focusing on negative results would likely be published in a lower impact journal and is therefore not worth my time	○	○	○	○	○
I have omitted negative results from a publication in order to make room for more impactful positive results	○	○	○	○	○

You're all done!

Thank you for participating in this research survey.

If you have any questions regarding the survey or this research project in general, please contact eb.labsurvey@gmail.com.

If you have any questions concerning your rights as a research participant, please contact the IRB of the University of Arizona (https://tinyurl.com/UAZIRB).

22. If you have any comments, concerns, or additional information pertaining to your unpublished data or unused samples, please share below.
